# Identification of co-expressed gene networks promoting CD8^+^ T cell infiltration and having prognostic value in uveal melanoma

**DOI:** 10.1186/s12886-023-03098-7

**Published:** 2023-08-10

**Authors:** Chun Zhang, Jing Xiao, Luzhong Fa, Fanwen Jiang, Hui Jiang, Lin Zhou, Zhuping Xu

**Affiliations:** Department of ophthalmology, West China Hospital, Sichuan University, Sichuan Province 610041 Chengdu, China

**Keywords:** CD8^+^ T cells, Weighted gene co-expression network analysis, Uveal melanoma, Immune microenvironment

## Abstract

Current immunotherapies are unsatisfactory against uveal melanoma (UM); however, elevated CD8^+^ T cell infiltration level indicates poor prognosis in UM. Here, we aimed to identify co-expressed gene networks promoting CD8^+^ T cell infiltration in UM and created a prognostic hazard model based on the identified hub genes. Raw data and clinical information were downloaded from The Cancer Genome Atlas (TCGA) and Gene Expression Omnibus (GEO) databases. Stromal-immune comprehensive score (ESTIMATE) was used to evaluate the immune-infiltration landscape of the tumor microenvironment. Single-Sample Gene Set Enrichment Analysis (ssGSEA) and Weighted Correlation Network Analysis (WGCNA) were used to quantify CD8^+^ T cell infiltration level and identify hub genes. Gene ontology (GO) and Kyoto Encyclopedia of Genes and Genomes (KEGG) enrichment analyses were performed to analyze the biological processes. Least absolute shrinkage and selection operator (LASSO) Cox regression were used to establish a prognostic model, which was further validated. Finally, pan-cancer analysis evaluated these genes to be associated with CD8^+^ T cell infiltration in other tumors. In conclusion, the proposed four-gene *(PTPN12*, *IDH2*, *P2RX4*, and *KDELR2*) prognostic hazard model had satisfactory prognostic ability. These hub genes may promote CD8^+^ T cell infiltration in UM through antigen presentation, and CD8^+^ T cell possibly function as Treg, resulting in poor prognosis. These findings might facilitate the development of novel immunotherapies.

## Introduction

Uveal melanoma (UM) is the most common primary intraocular malignancy in adults. It is an aggressive malignancy composed of malignant melanoma cells. More than 90% of UM involves the choroid and usually presents with symptoms such as blurred or distorted vision, visual field loss, or photopsia. UM has a strong tendency to metastasize from the eyes to the liver [1]. Approximately 40% of UM patients have liver metastases, leading to a high mortality rate [2]. Patients receiving UM treatment have a high rate of local tumor control, however, most patients eventually die of metastasis and have a median overall survival (OS) duration of 12 months [3].

The treatment of metastatic UM includes systemic chemotherapy, immunotherapy, and molecular targeted therapy [4]. Among them, immunotherapy has achieved remarkable results in the treatment of various malignant tumors. However, the low mutational burden and low immunogenicity of UM may result in high resistance to immunotherapy, and the response rate to anti-cytotoxic T lymphocyte antigen 4 (CTLA-4) and anti-programmed cell death-1 (PD-1) checkpoint inhibitor therapy is limited [5–7]. These immunotherapies cannot prolong the survival period of patients with metastatic UM [8]. Therefore, developing new immunotherapies for metastatic UM has become a priority.

Recently, a new immune-mobilizing monoclonal T-cell receptors (TCR) against cancer (ImmTAC), called Tebentafusp, has recently been confirmed to be the first drug to significantly prolong the survival of patients with metastatic UM [9]. The U.S. Food and Drug Administration (FDA) granted approval to tebentafusp-tebn (Kimmtrak) for the treatment of adult patients with HLA-A*02:01–positive, unresectable or metastatic uveal melanoma. As described in the FDA’s approval of Tebuconazole, this drug that pairs an anti-CD3 immune effector cell-binding domain with a high-affinity gp100-directed TCR is not an option for every patient with advanced uveal melanoma. Because the immune-mobilizing TCR portion of tebentafusp recognizes gp100 peptides presented only on the HLA-A*02:01 molecule, the coverage of this therapy is limited to 40% of individuals worldwide [9, 10]. Furthermore, although tebentafusp extended overall survival at 1 year to 73% and median survival to 21.7 months in metastatic UM patients, the prognosis of metastatic UM remains poor compared to other cancers, therefore, new immunotherapeutic approaches still need to be explored in order to further prolong survival time of patients [11].

As the main component of the tumor microenvironment (TME), immune infiltrates contribute to tumor progression and immunotherapy response [12]. Tumor infiltrating lymphocytes (TILs) are polymorphic, mainly including T, B, NK, and myeloid cells [13]. TILs in uveal melanoma are predominantly CD8^+^ T cells; the frequency of CD4^+^ T cells is low, and B cells and NK cells are only rarely identified [14–16]. In particular, CD8^+^ T cells play a central role in cancer immunity by recognizing specific antigenic peptides on the surface of target cells through the TCR, thus killing malignant cells [17]. Therefore, T lymphocyte infiltration in a variety of tumor biopsy samples has been associated with improved survival in a series of retrospective studies of patients with cancer [18–20]. However, several studies have shown that higher TIL infiltration and lower PD-L1 expression in UM appear to be associated with poor prognosis [21–23]. These findings are contrary to other studies where higher PD-L1 expression was correlated with poor outcome in solid tumors including cutaneous melanoma [24, 25]. It could be one of the reasons for the ineffectiveness of existing immunotherapies against UM. Therefore, identification of tumor-infiltrating CD8^+^ T cell-related genes may provide ideas for the development of new immunotherapies applicable to UM.

In this study, we used bioinformatic methods to identify co-expressed gene networks promoting CD8^+^ T cell infiltration in UM. Then, a prognostic hazard model based on the hub genes was constructed. We further analyzed the biological processes involved in these hub genes by GO, KEGG, and GSEA, and proposed new ideas for treating UM based on these genes and biological processes. This study could be helpful to achieve the goal of enhancing the efficacy of immunotherapy against UM.

## Materials and methods

### Data source

**Fig. 1:**
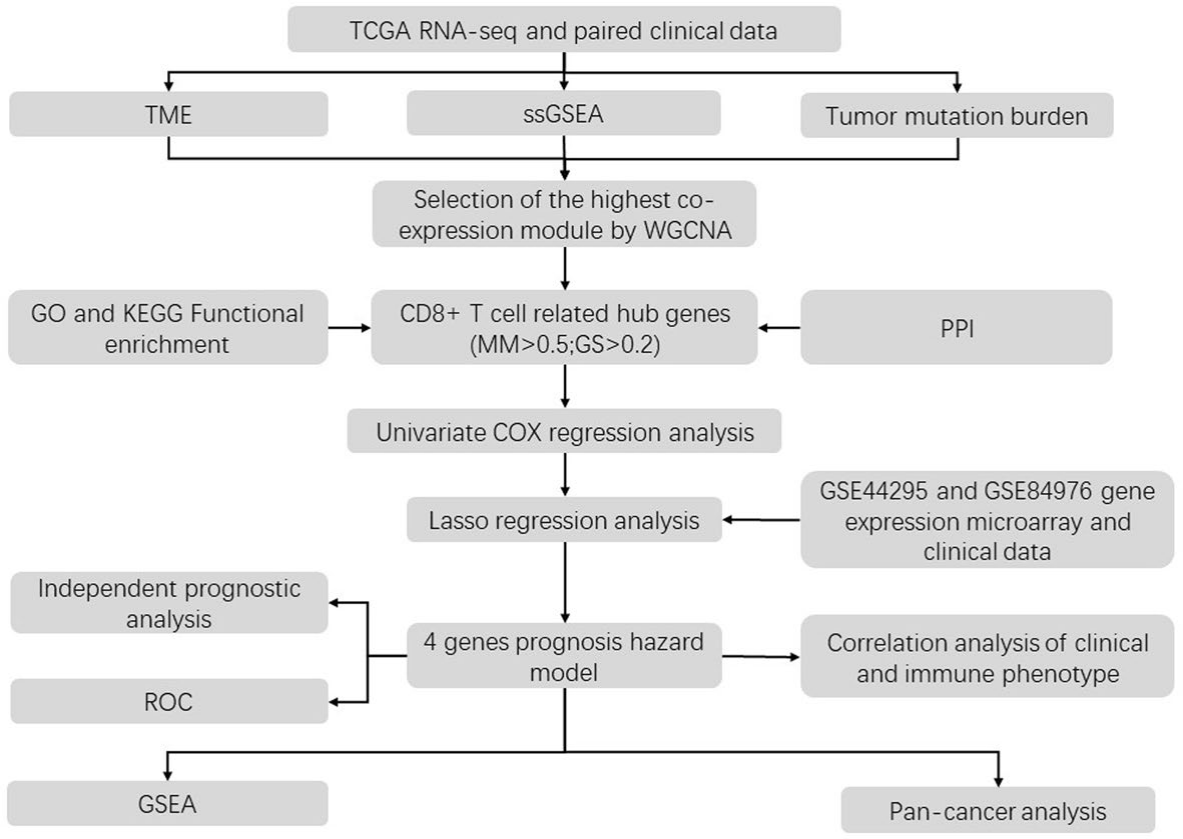
The work flow chart of this research

First, RNA sequencing (RNA-seq) and paired clinical data of UM were downloaded from the TCGA database (https://portal.gdc.cancer.gov/). The RNA-seq transcriptome data (in FPKM) were annotated using the human General Transfer Format (human.gtf) from the Ensembl database (https://www.ensembl.org/) with the Strawberry Perl software (version 5.28.2.1). Second, gene expression microarray and clinical data from the GSE44295 and GSE84976 dataset were downloaded as a series of matrix files from the Gene Expression Omnibus (GEO) (http://www.ncbi.nlm.nih.gov/geo/) database. Probe IDs were matched to gene symbols using the GPL6883 and GPL10558 platform. The mean expression value of the probes was used as the expression value for the gene in question if multiple probes were mapped to a single gene. A detailed work flow chart of this research displaying our entire analysis process is shown in Fig. 1. All methods were performed in accordance with the guidelines and regulations in the guidelines for analysis of RNA sequencing data published by Koch et al. and Chen et al. [26, 27].

### Immune cell infiltration analysis

The 29 immune-associated marker gene sets were obtained from a previous study [28]. The enrichment scores of the 29 immune-associated gene sets in each UM sample were quantified by single-sample gene set enrichment analysis (ssGSEA) using the “GSVA” R package. To identify gene signatures expressed by immune cell populations and immune pathways, ssGSEA was applied to every tumor sample.

### Tumor microenvironment (TME)

We obtained the TME of UM cases from the TCGA database. Immune (immune cell infiltration), stromal (stromal content), and stromal-immune comprehensive score (ESTIMATE) scores, as well as tumor purity were calculated for every UM sample using the ESTIMATE algorithm integrated in the “estimate” R package [29]. In addition, TCGA-UM SNP data were obtained, and the tumor mutation burden of each sample was evaluated.

### Weighted correlation network analysis (WGCNA)

WGCNA is a data mining method especially designed for studying biological networks based on pairwise correlations between variables. WGCNA was used to identify co-expression gene modules, explore associations between gene networks and CD8^+^ T cell infiltration levels. After WGCNA analysis, modules that are highly correlated with clinical features are defined as hub modules, and genes with high correlation in hub modules are hub genes [30, 31]. The soft threshold power of β was calculated following the scale-free topological criterion, and a weighted adjacency matrix was generated. The number of genes in the minimum module was set as 100. In addition, modules with a distance of less than 0.25 were merged. CD8^+^ T cell infiltration levels, stromal score, immune score, tumor purity, and TMB were used as input files for correlation analysis between modules and phenotypes. We identified the hub genes with module membership (MM) > 0.5 and gene significance (GS) > 0.2 in the co-expressed modules having the highest correlation with CD8^+^ T cell infiltration.

### Enrichment analysis and protein-protein interaction (PPI) network of hub genes

We converted the hub gene id to entrez ID and performed GO (http://geneontology.org/) enrichment analysis to explore the biological function, cell composition, and molecular function of the selected hub genes. Moreover, Kyoto Encyclopedia of Genes and Genomes (KEGG) enrichment analysis was performed to explore significant enrichment pathways. The recurring instances of neighboring genes (STRING) online search tool (version 11.5) was used to construct a PPI network (minimum required interaction score > 0.7) [32]. Then, Cytoscape software (version 3.9.0) was used to build the final PPI network [33].

### Construction of the gene-related prognostic model

Univariate Cox regression analysis was used to identify genes significantly related to prognosis, and least absolute shrinkage and selection operator (LASSO) COX regression analysis was applied to further narrow the scope of UM marker genes and create a prognostic hazard model by “glmnet” and “survival” R packages. Kaplan–Meier analysis and area under the curves (AUCs) were used to evaluate the accuracy of the prognostic hazard model in the TCGA-UM, GSE44295 and GSE84976 cohorts respectively. Finally, univariate and multivariate Cox regression analyses were used to evaluate whether the constructed prognostic hazard model can be used as an independent prognostic factor.

### Gene set enrichment analysis (GSEA)

The gene matrix in TCGA-UM was divided into low- and high-expression groups according to the median expression levels of hub genes, and the GSEA software (version 4.1.0) was used to determine the biological functions of these genes.

### Pan-cancer analysis

Tumor Immunity Estimation Resource (TIMER2.0; http://timer.cistrome.org/) was used to analyze the correlation between the derived hub genes in UM and CD8^+^ T cell infiltration levels in 32 additional cancers [34].

### Statistical analysis

All statistical analyses were performed using R version 4.1.1 software (http://www.r-project.org/) and Sangerbox tools (http://www.sangerbox.com/tool). Statistical significance was set at *p* < 0.05.

## Results

### Identification of CD8^+^ T cell infiltration-related modules in UM

**Fig. 2:**
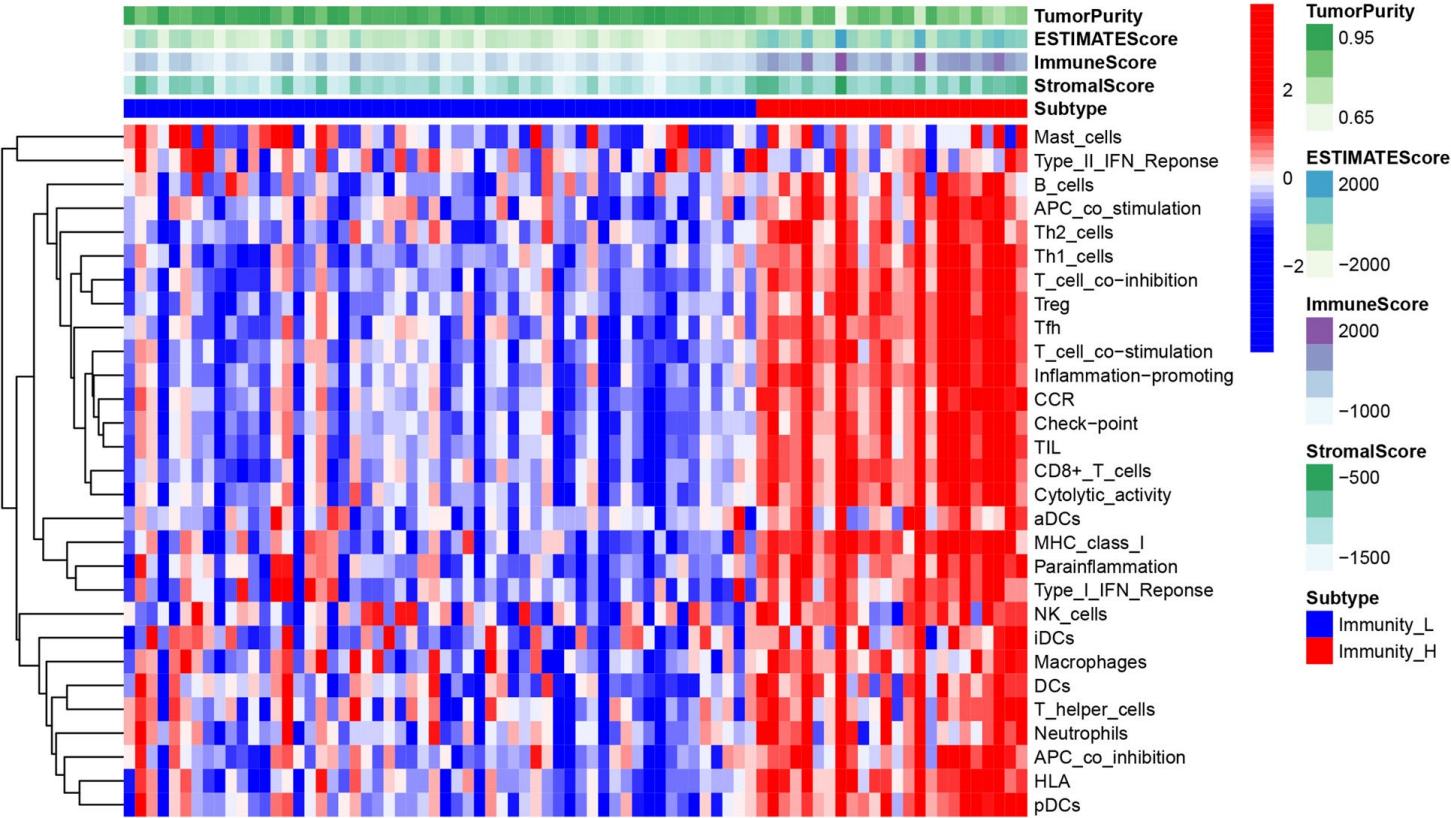
ssGSEA results, immune score, estimated score, stroma score, and tumor purity of samples in TCGA-UM.

**Fig. 3:**
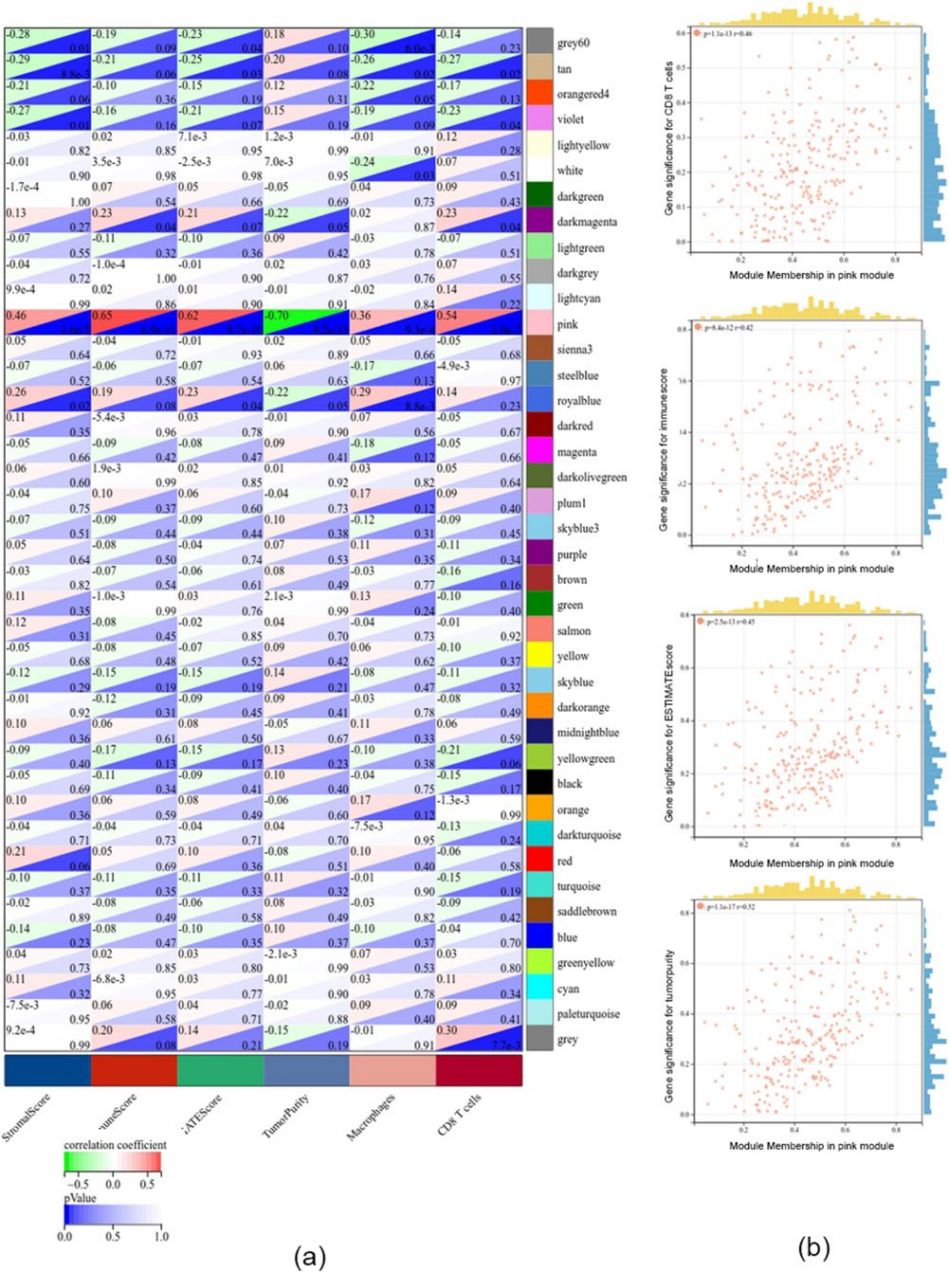
The result of WGCNA analysis. **a** Correlation between different phenotypes and co-expression modules. **b** Correlation of genes in pink modules with CD8^+^ T cell levels, immune score, estimated score, stroma score, and tumor purity

To quantify the levels of immune cell infiltration in the TCGA-UM cohort, we used ssGSEA to calculate the enrichment score for all immune cells in each sample. The TCGA cohort was divided into low- and high-immunity groups by applying unsupervised consensus clustering analysis. To validate the immunity of immune subgroups, we show the TME in the heatmap (Fig. 2). The high-immunity group had higher CD8^+^ T cell enrichment score and lower tumor purity. Then, WGCNA was performed to identify hub genes and co-expression modules with similar expression profiles related to CD8^+^ T cell infiltration in UM. To build a scale-free network, a value of β = 4 was identified as a soft threshold. We clustered the genes of 80 samples and constructed a hierarchical clustering tree using the dynamic hybrid cutting method, and finally generated 28 co-expression models. Each leaf on the tree represents a gene, and genes with similar expression patterns are grouped together to form a branch of the tree, representing a gene module. Finally, Spearman correlation analysis was used to evaluate the correlation between modules and phenotypes (Fig. 3a). Among them, the pink module containing 240 genes had the highest correlation with CD8^+^ T cell infiltration (r = 0.54) and was the most significant module (p = 2e-7); thus, it was selected as the hub module. These immune-associate phenotypes were defined as gene significance (GS). The MM and GS correction analyses of the pink module are shown in Fig. 3b. GS and MM were closely correlated in the pink module for UM tissue, reflecting the strong correlation between UM tissue and the pink module genes.

### Functional analysis of the CD8^+^ T cell-infiltration related hub Module

**Fig. 4:**
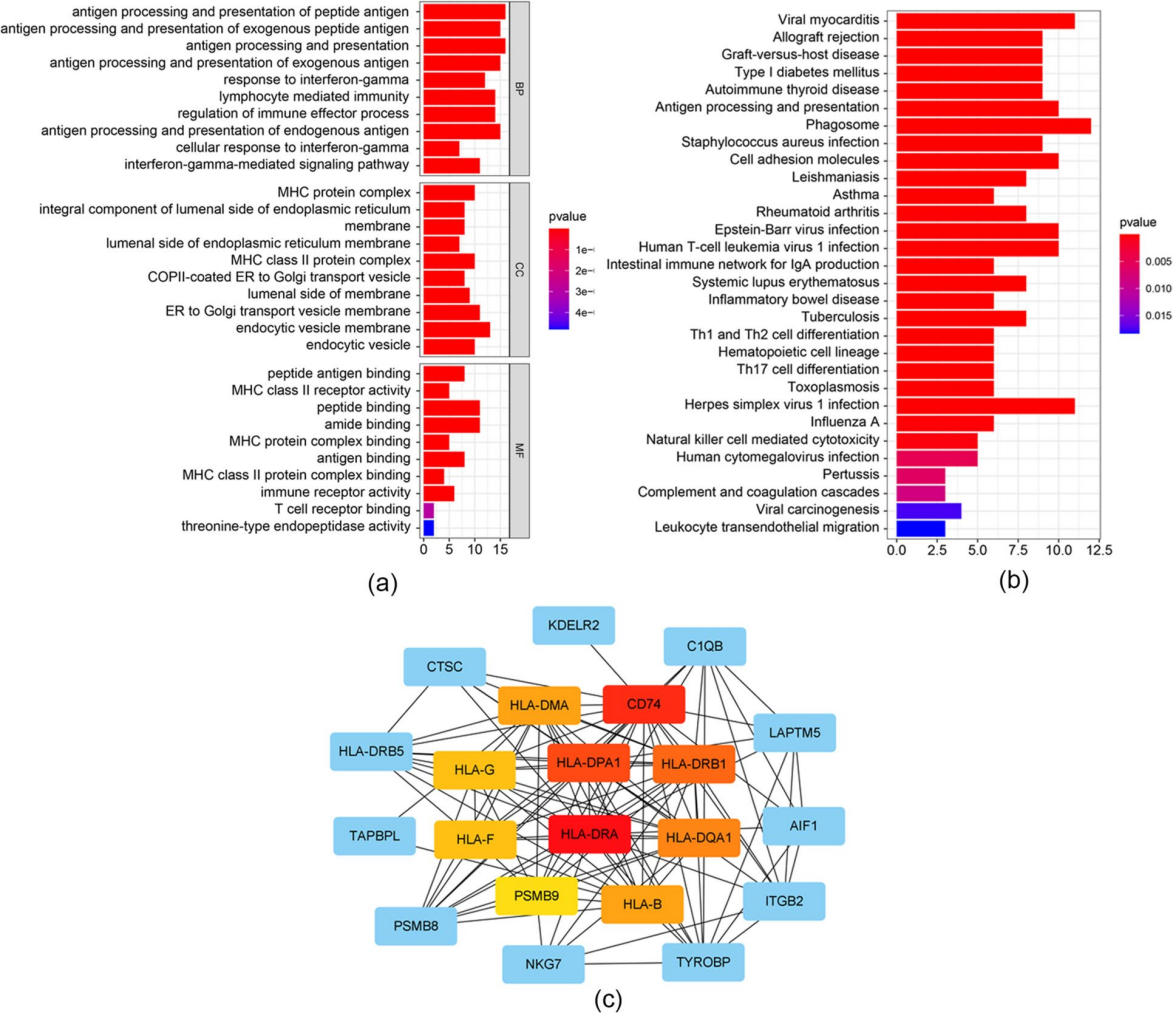
The result of GO and KEGG analysis (**a**) GO enrichment analysis of pink module; (**b**) KEGG enrichment analysis of pink module. **(c)** Protein-protein network of the pink module

To explore the underlying mechanism and biological functions of the CD8^+^ T cell infiltration-related module, 61 hub genes of the pink module were extracted for GO term and KEGG pathway annotation analyses. GO analysis identified “antigen processing and presentation of peptide antigen” as the most significant biological processes (Fig. 4a). KEGG analysis identified pathways significantly related to immunity, including antigen processing and presentation (Fig. 4b). To explore the interaction between hub genes, a PPI network was constructed using the STRING database and Cytoscape software (Fig. 4c).

### Construction of a prognostic hazard model

**Fig. 5:**
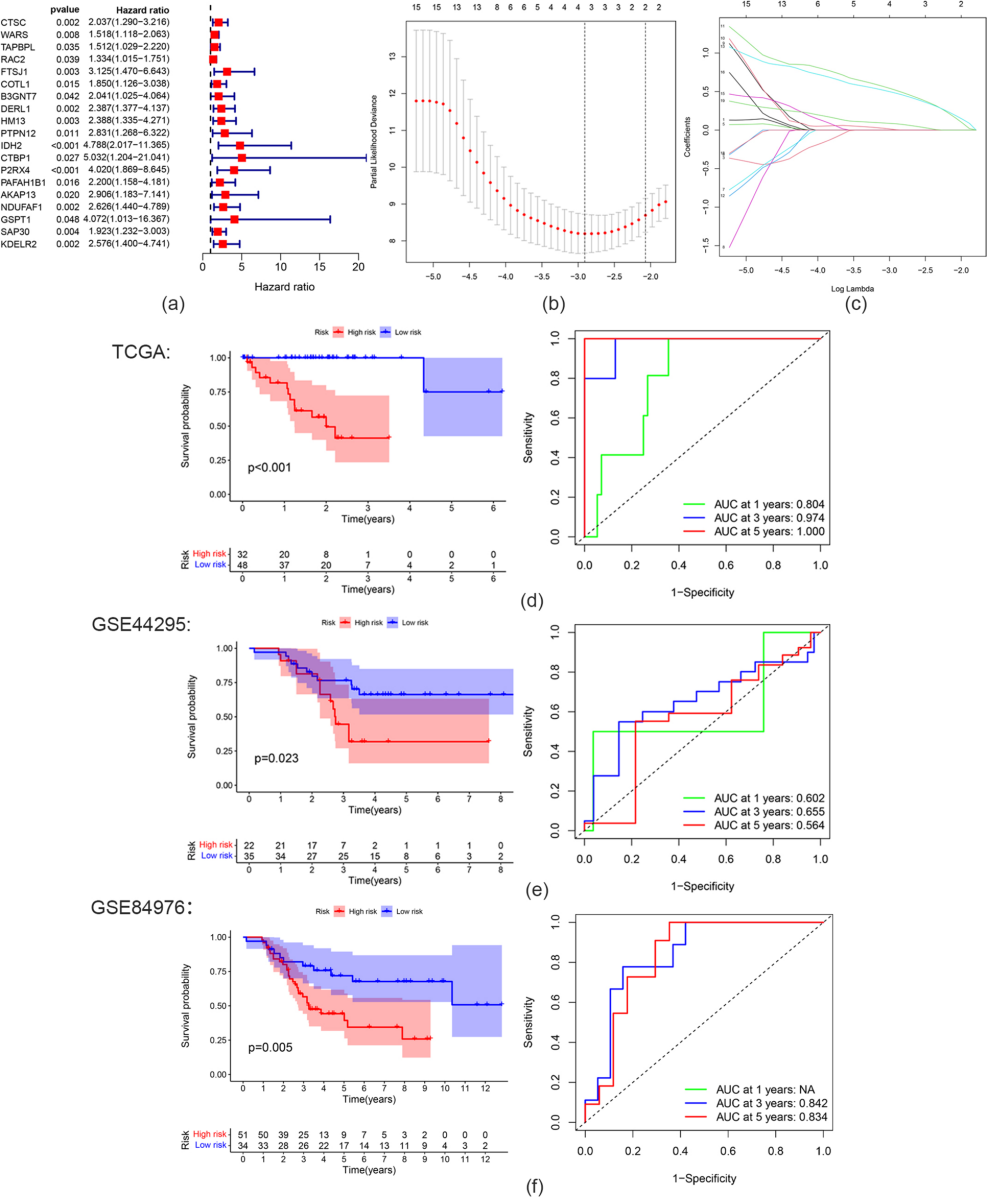
Construction and validation of prognostic hazard model. **a** Hazard ratio (HR), 95% confidence interval (CI) of prognostic-related genes calculated by univariate Cox regression. **b**, **c** Prognostic hazard model constructed by Lasso regression analysis. **d**–**f** Survival analysis and AUCs of TCGA and GEO data

**Fig. 6:**
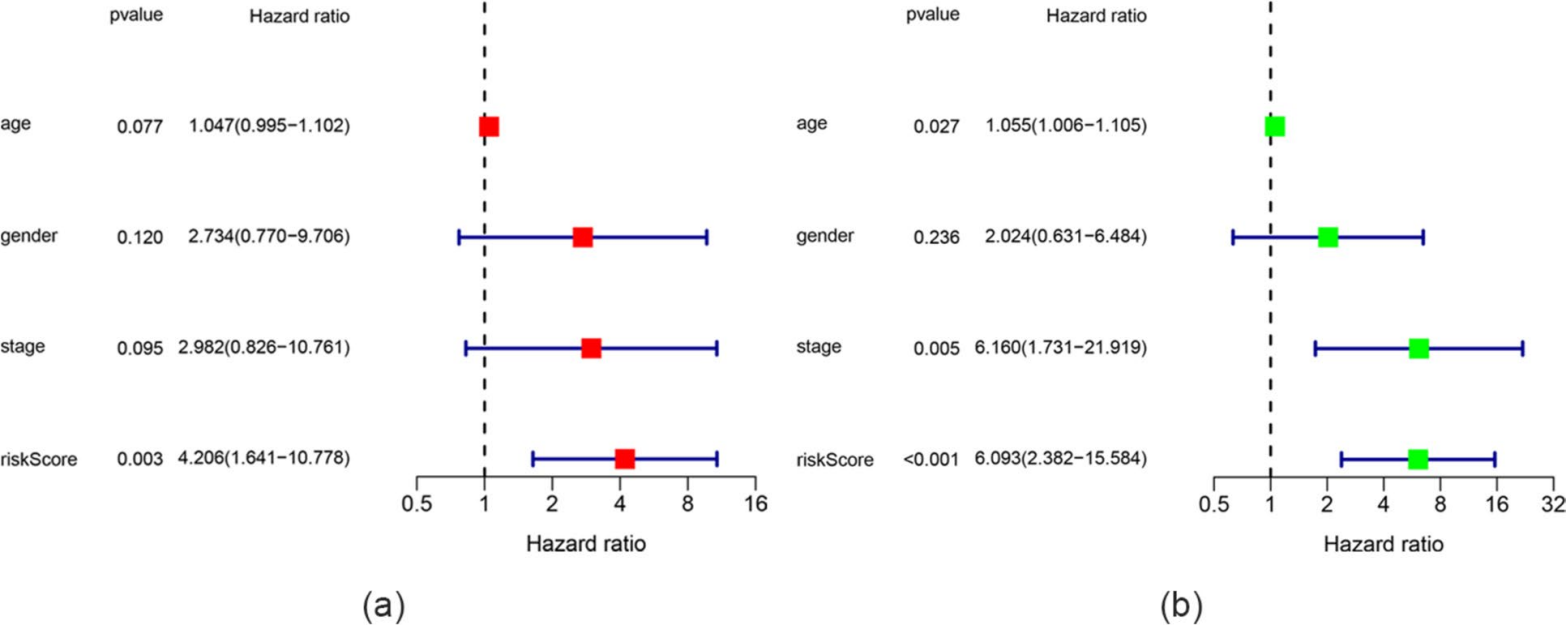
**a, b** Multivariate/Univariate Cox regression analyses of the association between clinical factors (including the risk score) and overall survival (OS) of patients

To further identify CD8^+^ T cell infiltration-related biomarkers that could predict UM prognosis, univariate Cox regression and LASSO Cox regression were used to analyze hub genes in the pink module. First, univariate Cox regression analysis was used to identify genes related to prognosis among the CD8^+^ T cell infiltration-related hub genes in the pink module (Fig. 5a). Then, Lasso Cox regression analysis based on 19 eligible genes (*p* < 0.05) was used to construct a prognostic hazard model (Fig. 5b, c). TCGA and GEO data were used as the training and test datasets, respectively. As a result, we obtained four prognostic genes for constructing the model. The risk score was calculated by summing the weighted gene expression level of each of the four genes multiplied by their respective LASSO coefficients: Risk score = 0.00399 * *PTPN12* + 0.51624 * *IDH2* + 0.47885 * *P2RX4* + 0.08205 * *KDELR2*. The constructed hazard model was used to calculate the risk score of every sample, and the samples were divided into high- and low-risk groups according to the Youden index. The prognosis of the high-risk group was significantly worse than that of the low-risk group (*p* < 0.001). In addition, AUCs were drawn to assess the prognostic power of the risk characteristics. The AUCs of the prognostic hazard model corresponding to 1-, 3-, and 5-year overall survival (OS) were 0.808, 0.974, and 1.000, respectively, which demonstrates the high accuracy of this model (Fig. 5d). As shown in Fig. 5e,f, the OS of the low-risk group from the GEO dataset was significantly higher than that of the high-risk group (p = 0.023, p = 0.005). This result is consistent with our previous findings in the TCGA cohort. This four-gene prognostic hazard model corresponds to AUCs of 0.602, 0.655 and 0.564 for 1-year, 3-year and 5-year OS in GSE44295 and 0.842 and 0.834 for 3-year and 5-year OS in GSE84976, respectively, which further confirms that this model has high sensitivity and specificity and can be used as a reliable predictor of OS in patients with UM. Finally, univariate Cox regression analysis showed that age, stage, and risk score had prognostic value, but gender did not. Then, multivariate Cox regression analysis showed that only the risk score can be used as an independent prognostic factor (Fig. 6). Therefore, the risk score can be used as a prognostic factor of patient OS independent of other clinical characteristics.

### Correlation analysis of clinical and immune phenotype using the four model genes

**Fig. 7:**
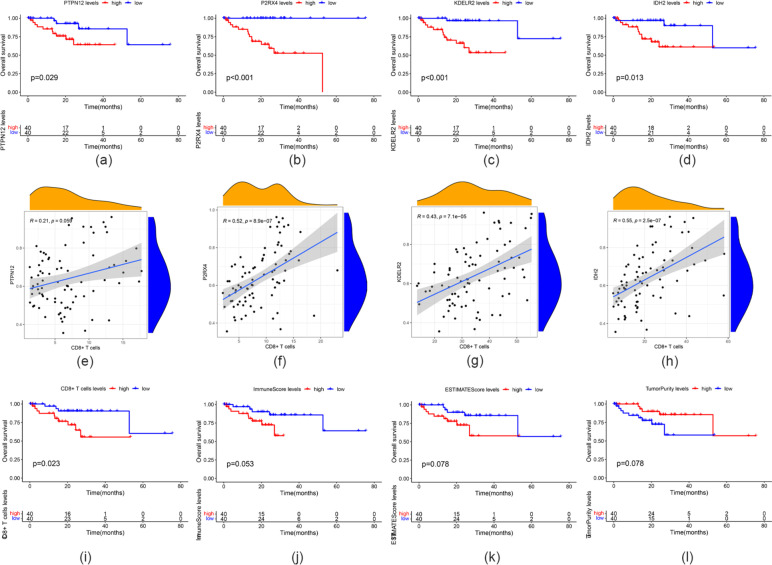
Relationship of model genes with OS, CD8^+^ T cell levels, and TME. **a–d** Survival analysis using PTPN12, IDH2, P2RX4, and KDELR2. **e–h** The correlation of PTPN12, IDH2, P2RX4, and KDELR2 expression with CD8^+^ T cell levels. **i–l** Survival analysis using the CD8 + T cell level, immune score, ESTIMATE score, and tumor purity

**Fig. 8:**
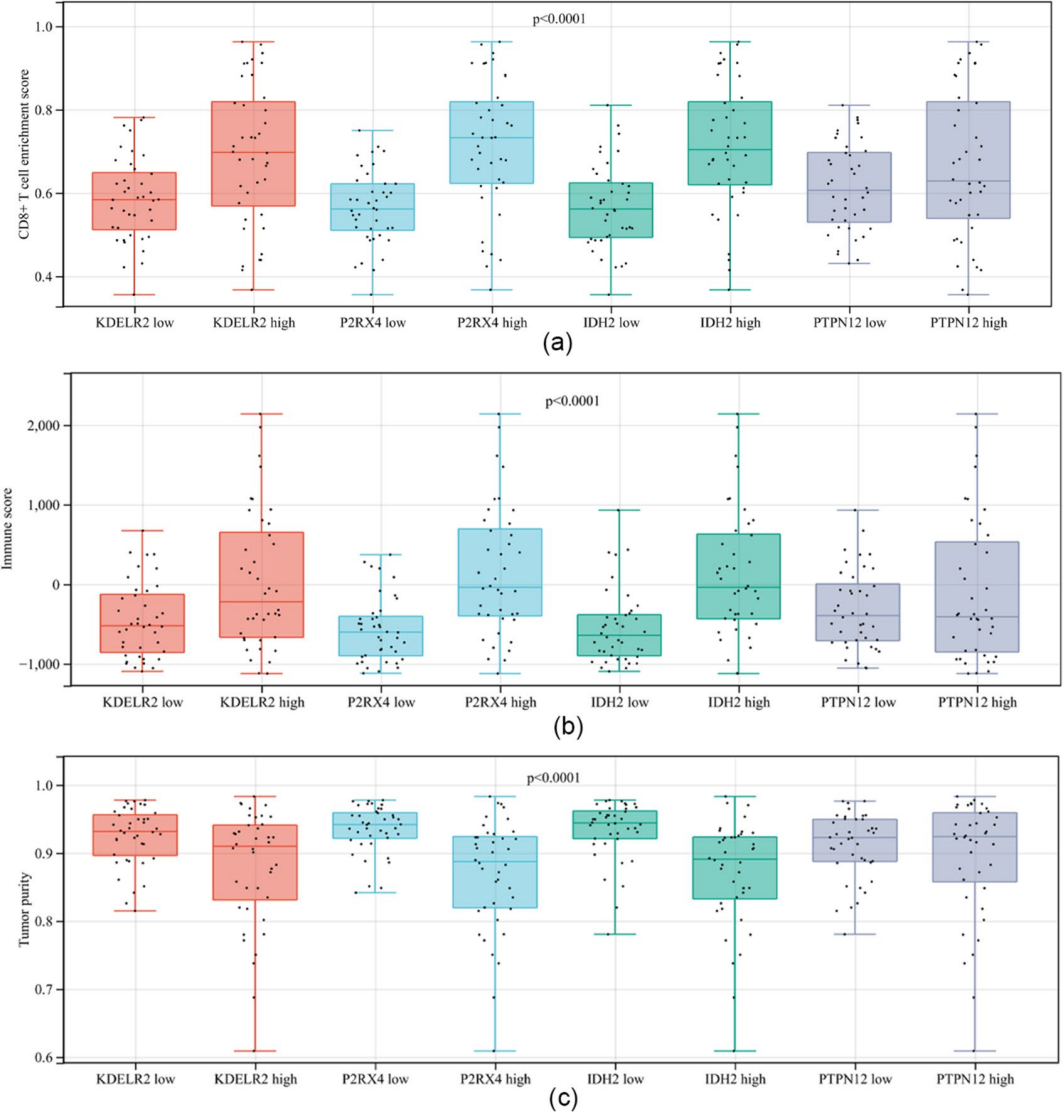
**a** Phenotype analysis of modular genes with CD8^+^ T cell enrichment score. **b** Phenotype analysis of modular genes with immune score. **c** Phenotype analysis of modular genes with tumor purity

To explore the relationship between the four model genes and clinical and immunophenotypes, the TCGA-UM cohort was divided into high- and low-expression groups according to the expression levels of these genes, and survival analysis was performed to examine their relevance to clinical outcomes. The patients in the high-expression groups for *PTPN12* (TCGA: p = 0.029), *IDH2* (TCGA: p = 0.013), *P2RX4* (TCGA: *p* < 0.001), and *KDELR2* (TCGA: *p* < 0.001) showed survival risk against low expression groups (Fig. 7a–d). We also found that the expression of these genes was positively correlated with the levels of CD8^+^ T cells (Fig. 7e–h). Moreover, patients with low levels of CD8^+^ T cell infiltration had longer OS (Fig. 7i–l). To determine more specifically the relationship between these genes and clinical phenotypes and immunophenotypes, we drew multiple sets of box plots. The CD8^+^ T cell infiltration level and immune score in the high-expression groups of these four genes were higher than those in the low-expression groups. In contrast, tumor purity was lower in the low-expression groups (Fig. 8a–c). These results suggest that the expression of the four genes may lead to poor prognosis by promoting CD8^+^ T cell infiltration.

### GSEA

**Fig. 9:**
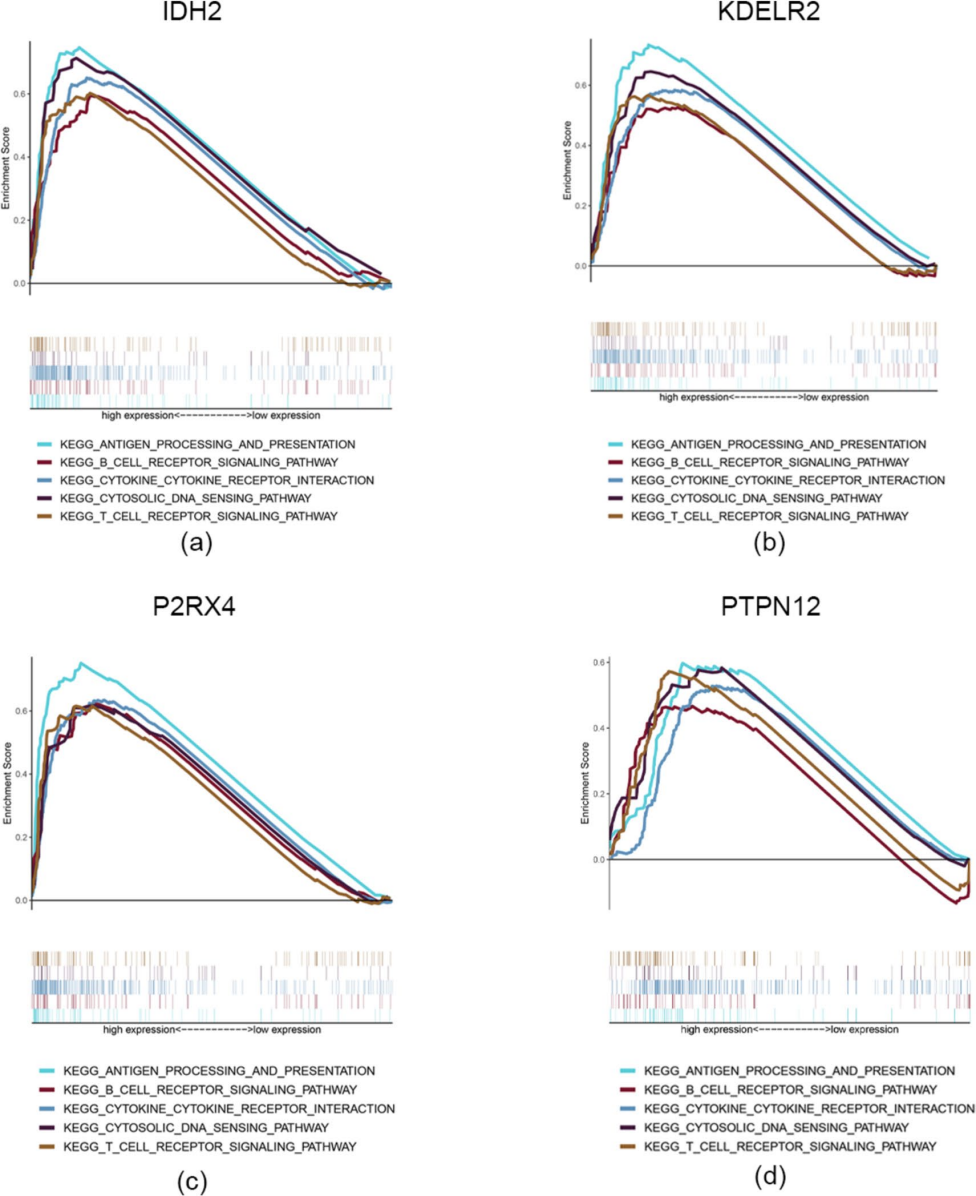
GSEA: **a–d** The results of immune-related GSEA analysis

The results of GSEA showed that high *PTPN12*, *IDH2*, *P2RX4*, and *KDELR2* expression was significantly related to immune-related pathways such as antigen processing and presentation, chemokine signaling, cytokine-cytokine receptor interaction, T cell receptor signaling, and B cell receptor signaling (Fig. 9a-d). These pathways are also involved in tumor immunity by protecting against tumor infiltration, thus providing a reference for exploring the mechanism whereby hub genes lead to poor prognosis by promoting CD8^+^ T cell infiltration.

### Pan-cancer analysis

**Fig. 10:**
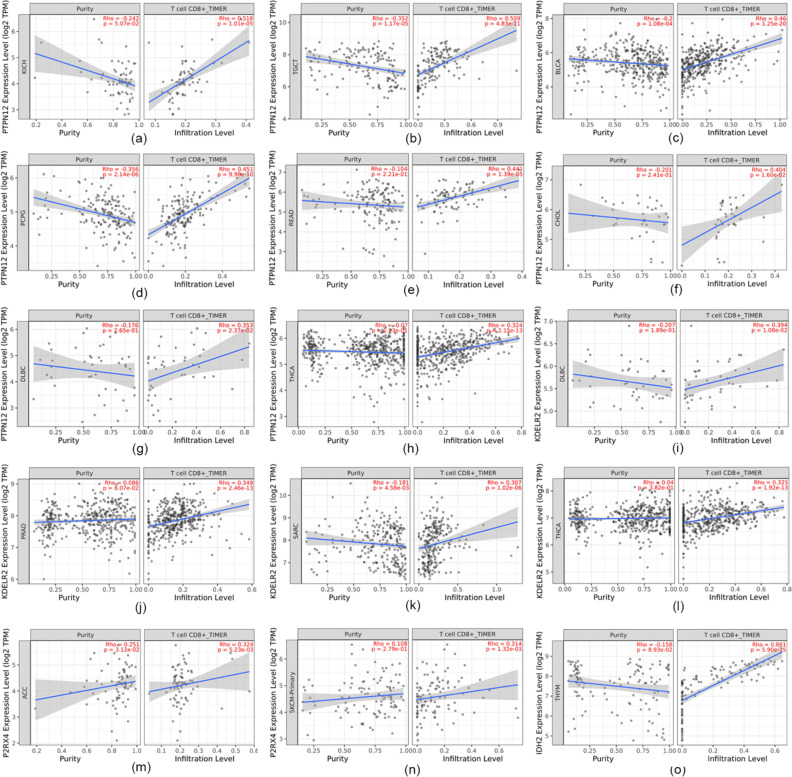
Pan-cancer analysis. **a–o** Pan-cancer analysis results of PTPN12, IDH2, P2RX4, and KDELR2, and correlation analysis of KICH (Kidney Chromophobe), TGCT (Testicular Germ Cell Tumors), BLCA (Bladder Urothelial Carcinoma), PCPG (Pheochromocytoma and Paraganglioma), READ (Rectum adenocarcinoma), CHOL (Cholangio carcinoma), DLBC (Lymphoid Neoplasm Diffuse Large B-cell Lymphoma), THCA (Thyroid carcinoma), PRAD (Prostate adenocarcinoma), SARC (Sarcoma), ACC (Adrenocortical carcinoma), SKCM (Skin Cutaneous Melanoma), THYM (Thymoma), and CD8^+^ T cells

The above results demonstrated the role of *PTPN12*, *IDH2*, *P2RX4*, and *KDELR2* in UM. To explore whether *PTPN12*, *IDH2*, *P2RX4*, and *KDELR2* can promote CD8^+^ T cell infiltration in other types of tumors, TIMER2.0 was used to analyze their correlation. As shown in Fig. 10, these four genes were also related to the level of CD8^+^ T cell infiltration in some tumors. Among thirty-six tumor types excluding uveal melanoma, PTPN12 was associated with CD8^+^ T cell infiltration in eight tumors including bladder urothelial carcinoma (BLCA), cholangio carcinoma (CHOL), and lymphoid neoplasm diffuse large B-cell lymphoma (DLBC); KDELR2 was associated with CD8^+^ T cell infiltration in four tumors including DLBC, rectum adenocarcinoma (READ), and sarcoma (SARC); P2RX4 was associated with CD8^+^ T cell infiltration in adrenocortical carcinoma (ACC) and skin cutaneous melanoma (SKCM); IDH2 was only associated with CD8^+^ T cell infiltration in thymoma (THYM).

## Discussion

Owing to its high mortality, UM has brought enormous socioeconomic pressure to patients and their families. Immune checkpoint inhibitors (ICIs) offer a new therapeutic strategy, ICIs are usually antibodies that block proteins expressed on specific immune system cells (especially T cells) and tumor cells. These antibodies provide checkpoints that help keep the immune response from becoming too strong. Blocking these checkpoints allows T cells to be more powerful in killing cancer cells. The most widely used targets of ICIs are CTLA-4 and PD-1 [13]. Although these antibodies are effective in treating cutaneous melanoma (CM), they are less effective in treating UM [5, 6]. This may be due to the fact that the eye is an immune-privileged site that limits the onset of immune-mediated inflammation. Furthermore, the lack of lymphatic drainage in the eye allows the retention of tumor antigens in the ocular environment, ultimately leading to CD8^+^ T cell exhaustion because of continuous exposure [15, 35]. In addition, the mutational burden of UM is particularly low, unlike CM, which is known for its very high mutational burden, mostly because the skin is exposed to ultraviolet radiation, which may also account for the limited efficacy of ICIs against UM [36]. Although many immunotherapies have not achieved satisfactory efficacy in metastatic UM, with the advent of tebentafusp and improved awareness of the tumor microenvironment by researchers, immunotherapy continues to show great potential for UM.

In this study, we sought to identify co-expressed prognostic related genes that promote CD8^+^ T cell infiltration, which may help reveal the biological processes most closely related to CD8^+^ T cell infiltration. After using ssGSEA and ESTIMATE to evaluate the level of CD8^+^ T cell infiltration and TME in each tumor sample, WGCNA was used to construct a CD8^+^ T cell-related co-expression network and identity the hub module. Enrichment analysis showed that the genes in this module are mainly related to antigen processing and presentation. Moreover, a prognostic hazard model consisting of four genes (*PTPN12*, *IDH2*, *P2RX4*, and *KDELR2*) was constructed based on prognostic-related genes. These genes showed satisfactory clinical phenotype and immunophenotype correlation. Interestingly, all these genes are associated with higher CD8^+^ T cell infiltration and tumor progression. Finally, we evaluated the association between these four genes expression and CD8^+^ T cell infiltration in the other thirty-six kinds of tumors by pan-cancer analysis and found that PTPN12, KDELR2, P2RX4 and IDH2 were associated with CD8^+^ T cell infiltration in eight, four, two and one tumor types, respectively.

**Fig. 11:**
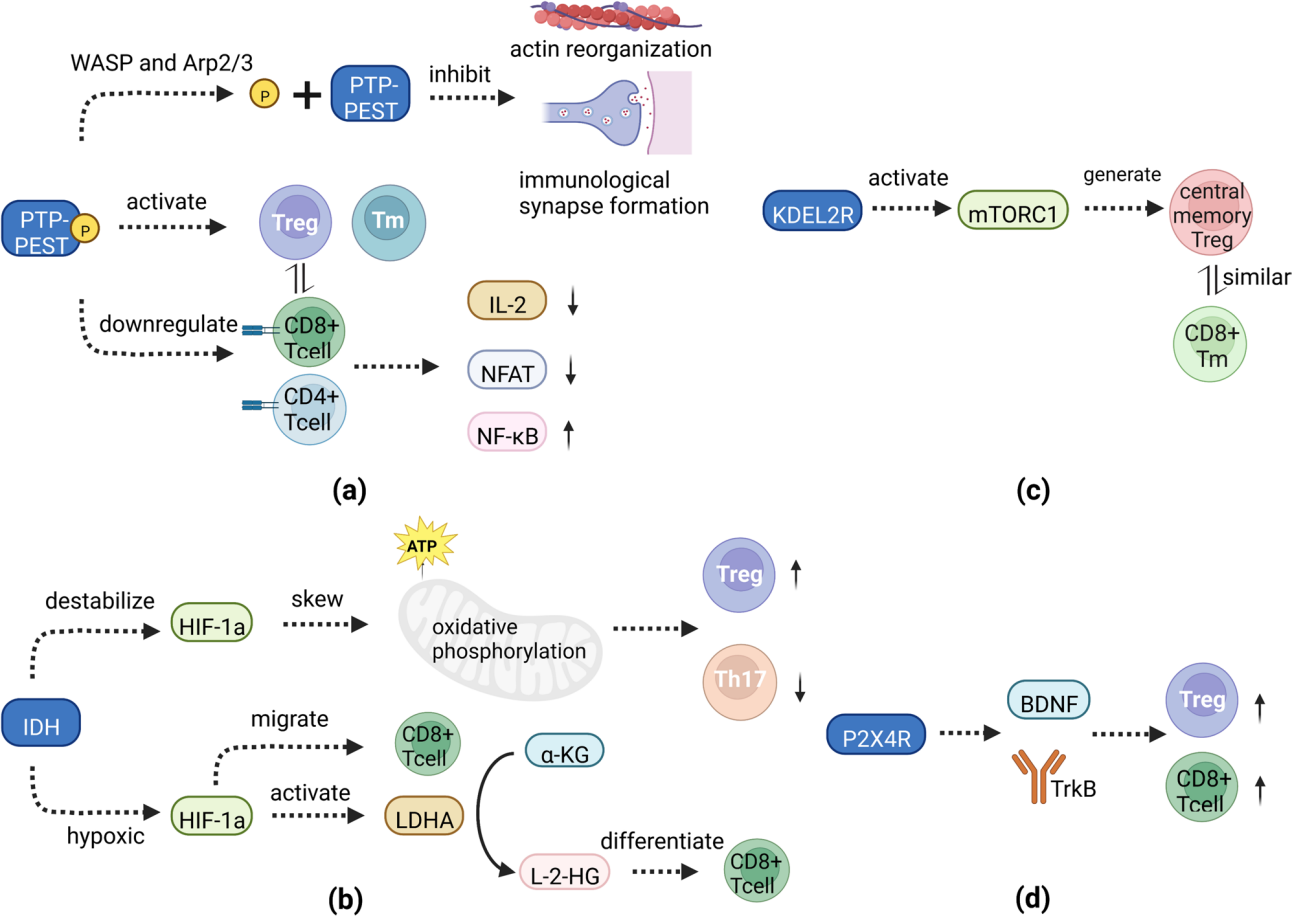
**a-d** Signaling pathways may associated with CD8^+^ T cell and Treg infiltration by four prognostic model genes, PTPN12, IDH2, P2RX4, and KDELR2.

PTP-PEST is an 88 kDa protein tyrosine phosphatase (PTP) encoded by *PTPN12* and containing a C-terminal PEST motif [37]. PTP-PEST serves as a protein-protein interaction domain that may regulate the intracellular half-life of proteins. Harris et al. showed that *PTPN12* protect tumor cells from abnormal reactive oxygen species accumulation and oxidative stress-induced death, thereby facilitating tumor cell development [38]. A study conducted by Weidemann et al. also demonstrated that elevated *PTPN12* expression parallel the tumor development and progression of prostate cancer [39]. Simultaneously, the expression of *PTPN12* in immune cells is higher than that in non-immune cells [40]. In *PTPN12* overexpressed-ovalbumin (OVA)-specific CD4^+^ T cells, *PTPN12* inhibits actin reorganization and immunological synapse formation through the dephosphorylation of WASP and Arp2/3 associated signaling proteins [41]. *PTPN12* also downregulates TCR-mediated activation of CD4^+^ and CD8^+^ T cells in primary human T cells and Jurkat cells, leading to decreased production of interleukin-2, production and inhibition of nuclear factor of activated T cells, nuclear factor kappa B [42]. In addition, downregulation of *PTPN12* leads to the enhanced responsiveness of secondarily activated T cells including effector and memory T cells [42]. This contradicts our finding that *PTPN12* promotes CD8^+^ T cell infiltration in UM, further suggesting that the role of infiltrating CD8^+^ T cells in UM may differ dramatically from other tumors. Therefore, it becomes particularly important to further explore the unique mechanism of *PTPN12* action on T cells and their signature cytokines in UM, and PTP-PEST inhibitors would be a promising candidate for UM immunotherapy (Fig. 11a).

IDH2 encodes an isocitrate dehydrogenase (IDH) isoforms that is a key metabolic enzyme catalyzing the interconversion of isocitrate to α-ketoglutarate (α-KG). Frequent mutations of IDH1 and IDH2 have been found in various tumors, such as acute myeloid leukemia (AML), glioma, and chondrosarcoma [43–45]. Tumor-associated IDH1 and IDH2 mutations occur almost exclusively at different arginine residues in the active enzyme site [46]. The mutation site of the IDH1 gene is located at R132 and that of the IDH2 gene at R140 and R172 [47]. The IDH mutation results in a decrease in its normal enzyme catalytic activity and a corresponding decrease in the production of the reactants NADPH and α-KG [47]. However, it acquires the new enzymatic activity that catalyzed the irreversible conversion of NADPH and α-KG into D-2-hydroxyglutarate (D-2-HG), which can competitively inhibit α-KG-dependent enzymes and subsequently induce cellular metabolic reprogramming, inhibit cell differentiation, and lead to cell tumorigenesis [47]. In addition, D-2-HG triggers hypoxia-inducible transcription factor 1 (HIF-1α) protein destabilization resulting in metabolic skewing towards oxidative phosphorylation, increased Treg frequency, and reduced T helper 17 (Th17) polarization [48]. The reduction in α-KG leads to DNA hypermethylation and reduced chromatin accessibility, which limits Treg-related functional gene expression and repressive activity [49]. Under hypoxic environments, HIF-1α promotes the migration of CD8^+^ T cells into tumor tissues [50, 51], and its activation leads to an increase in lactate dehydrogenase A, which converts α-KG to L-2-HG [52, 53]. Increased L-2-HG levels change the gene expression and differentiation pathway of CD8^+^ T cells and enhance the anti-tumor ability [54] (Fig. 11b). Therefore, inhibition of IDH mutation and promotion of α-KG conversion can reduce Treg percentage and thus inhibit tumor survival.

The protein encoded by *KDEL2R*, a member of the KDEL (lys-aspp-glu-leu) receptor (KDELR) family, is mainly responsible for extracting soluble endoplasmic reticulum proteins from the Golgi complex to maintain dynamic equilibrium transport [55]. In addition, it plays a key role in cell secretory trafficking, cell growth, autophagy, immune response, and tumorigenesis [56–60]. The regulation of Golgi secretion by *KDEL2R* is necessary for cell invasion and metastasis, and *KDEL2R* inhibition can reduce lung cancer metastasis [61]. Furthermore, *KDEL2R* promotes the occurrence of glioblastoma by regulating the phosphorylation level of mTOR (Ser2448). And *KDEL2R* could activate the mTORC1 pathway associated with cell proliferation and invasiveness [62]. The study of Sun et al. demonstrated that inhibition of mTOR during T cell activation promotes the generation of central memory Tregs in mice, these central memory Tregs possess enhanced spare respiratory capacity, similar to CD8^+^ memory T cells, the generation of effector Tregs also requires mTOR (Fig. 11c) [63]. Wei et al. also found that *KDEL2R* accelerates the cell cycle and promotes the progression of breast cancer by binding to and stabilizing the centrosome protein POC5 [64].

The purinergic receptor P2 × 4 (P2 × 4R) is encoded by *P2RX4* and belongs to the purinergic receptor family for ATP. This receptor functions as a ligand-gated ion channel with high calcium permeability. Huo et al. found that P2 × 4R depletion can inhibit tumor cell survival and proliferation and promote cell apoptosis [65]. Brain derived neurotrophic factor (BDNF) is a member of the neurotrophin superfamily, BDNF and its receptor TrkB plays a role in a variety of cancers [66–68]. In patients with stroke, the patient who had the high levels of CD8^+^ BDNF^+^ T cells had the highest levels of BDNF^+^ Tregs [69]. Therefore, perhaps by silencing P2 × 4R, the expression of BDNF/TrkB can be blocked and the proportion of Treg reduced, thus inhibiting the growth of UM tumor cells (Fig. 11d).

Unlike in other tumors, we found that high CD8^+^ T cell infiltration is a poor prognostic marker in UM. Previous studies suggested that tumor-infiltrating CD8^+^ T cells may function as Tregs, an important factor contributing to ocular immune privilege, which is the result of anterior chamber-associated immune deviation (ACAID) when antigens are introduced into the anterior chamber. This induces the appearance of CD8^+^ Tregs to inhibit Th1- and Th2-related immune responses [70, 71]. In addition, CD8^+^ Tregs can inhibit immune responses directly by killing immune cells, or indirectly by producing immunosuppressive molecules such as TGFβ and indoleamine 2,3-dioxygenase (INDO) [72]. Thus, CD8^+^ Tregs in UM promote tumor evasion of immune responses and lead to a poor prognosis. The intra tumoral/peritumoral ratio of CD8^+^ Granzyme B^+^ cells and density of intra-tumoral CD8^+^ CTLs is higher in patients with disease control than in those with progressive disease, revealing the importance of elevating tumor-killing T cells in UM. In our study, four genes associated with CD8^+^ T cell and Treg infiltration were identified, as shown in Fig. 11, regulating the activation or suppression of CD8^+^ T cells and Treg through novel signaling pathways. Based on these newly identified genes and biological processes, which could contribute to the development of new UM immunotherapeutic agents.

Our study demonstrated the critical role of CD8^+^ T cell-mediated immune infiltration in the poor prognosis of uveal melanoma. Besides, Skalet et al. identified a population of circulating tumor cells known as circulating hybrid cells (CHCs) in the peripheral blood of uveal melanoma patients, and this hybrid cell, which combines the characteristics of both tumor cells and macrophages, may play an indispensable role in tumor progression and metastasis, with the potential to serve as a non-invasive biomarker for uveal melanoma. CHC that diffuse into the circulation provide a suitable environment for the development of primary tumors, thus driving the metastatic process. The study by Skalet et al. also identified HTR2B as an enhanced CHC detection marker to identify high-risk uveal melanomas in combination with gp100 for improved detection [73]. Additionally, the presence of dual nature cells (DNC) with tumor and leukocytic cell features, which may correspond to CHC in the blood, was identified in UM samples. The investigators found that DNC were present in 63% of UM cases and that inflammation, which represents a poorer prognosis for patients, was present in these cases [74]. Our study identified that PTPN12, IDH2, P2RX4, and KDELR2 can influence the prognosis of UM by prompting the CD8^+^ T cell infiltration. Further exploration of the effects of these four genes on hybrid cells may help to further reveal the secrets of UM pathogenesis. In addition, both hybrid cells and CD8^+^ T cells are significant prognostic markers for UM, so it is necessary to explore the interaction between these two types of cells and tumor cells.

Overall, the effect of PTPN12, IDH2, P2RX4, and KDELR2 on tumor immune cell infiltration is a complex process that typically involves multiple molecular pathways and signaling pathways. The tyrosine phosphatase encoded by PTPN12 is involved in signal transduction pathways that regulate immune cell activation and infiltration [75]. Variants of PTPN12 in tumors may affect the recognition and infiltration capacity of immune cells and thus the infiltration of CD8^+^ T cells. Variations in KDELR2 may indirectly affect CD8^+^ T cell infiltration by affecting intracellular protein transport and metabolic pathways [57]. The protein encoded by P2RX4 plays an important role in immune cell activation and infiltration as a member of the ATP-gated channel family [76]. Variations in P2RX4 may lead to aberrant activation or inhibition of signaling pathways, which may affect CD8^+^ T cell infiltration. Variations in IDH2, which encodes an IDH involved in cellular energy metabolism and oxidative stress responses, may affect cellular metabolic and immunoregulatory pathways, leading to alterations in the tumor microenvironment, which in turn affects CD8^+^ T cell infiltration [77, 78]. Aberrant gene expression or mutation also affects the immune response, tumor microenvironment, and immune escape mechanisms of the tumor, which influence immune cell infiltration. Different tumor types have different gene variants and expression profiles, and therefore, as shown by the results of our pan-cancer analyses, these genes are only associated with CD8^+^ T cell infiltration in specific types of tumors, but not in all tumors. The effect of these genes on CD8^+^ T cell infiltration in tumors should be judged depending on factors such as tumor type, genetic mutation profile, tumor microenvironment, and patient characteristics. More in-depth research and analysis are still needed in the future to understand the correlation between these genes and tumor CD8^+^ T cell infiltration.

This article has some limitations. First, we used the TCGA-UM, GSE44295, and GSE84976 datasets for joint analysis. Although this is all the UM cohort we could find that contained prognostic information, more external cohorts need to be added for cross-validation. Second, we hypothesized that these genes can lead to poor immunotherapy efficacy by promoting the infiltration of CD8^+^ T lymphocytes; however, the efficacy of immunotherapy was not included in the TCGA-UM follow-up data and therefore, more immunotherapy follow-up data need to be added. Finally, we only discussed the role of these genes in tumor development, and the specific mechanism needs further study.

In conclusion, we constructed and verified a prognostic hazard model composed of the four genes, *PTPN12*, *IDH2*, *P2RX4*, and *KDELR2*, based on a CD8^+^ T cell infiltration co-expression network. These four co-expressed genes mainly promote CD8^+^ T cell infiltration by enhancing antigen processing and presentation, and their expression leads to a poor prognosis. This study can potentially provide novel biomarker and therapeutic targets for UM.

## Data Availability

The datasets analyzed during the current study are available in TCGA database (The Cancer Genome Atlas, http://cancergenome.nih.gov/), and GEO database (Gene Expression Omnibus database, https://www.ncbi.nlm.nih.gov/geo/).
